# Solitary metastasis of ampullary carcinoma to the spleen: a case report

**DOI:** 10.1002/ccr3.1414

**Published:** 2018-02-07

**Authors:** Tri M. Phan

**Affiliations:** ^1^ Department of Surgery University of Medical and Pharmacy at Ho Chi Minh City 217 Hong Bang Ho Chi Minh City Vietnam; ^2^ Department of Hepatobiliary and Pancreatic Cho Ray Hospital Ho Chi Minh City Vietnam

**Keywords:** Ampullary cancer, oligometastasis, pancreatoduodenectomy, splenic metastasis

## Abstract

Here, we report a first case of ampullary cancer with solitary metastasis of the spleen, which was successfully treated with pancreatoduodenectomy and splenectomy and was discharged 7 days after the operation with outpatient chemotherapy. In such cases, physicians should consider splenectomy as an effective treatment option.

## Introduction

Ampullary cancer accounts for about only 0.2% of malignant tumors of the intestine. Most ampullary cancers are carcinomas, such as adenocarcinoma (65%), carcinoma (8.1%), and other rarer types such as papillary adenocarcinoma (5.6%), mucinous adenocarcinoma (4.7%), and signet‐ring cell carcinoma (2%) 1, 2, 3, 4. Over half of ampullary cancer cases involve lymphatic metastasis; however, to the best of our knowledge, there have been no reports of ampullary cancer metastasized to the spleen. Splenic metastasis itself is very rare with approximately 7% of all splenic tumors originating from primary tumor metastasis 5, 6. In previous reports, most primary tumor locations in malignant secondary tumor of the spleen were breast, lung, and skin 1, 5, 7; however, we encountered a case in which the primary tumor location was the ampulla of Vater. Preoperative diagnosis to distinguish primary or secondary splenic tumor is difficult. Likewise, for total tumor removal, preoperative decisions such as the treatment goals, surgical how to approach, and postoperative care are problematic.

In this report, we describe a case of solitary splenic tumor metastasized from ampullary carcinoma that was successfully treated with pancreatoduodenectomy and splenectomy.

## Case Presentation

A 65‐year‐old woman, of an emaciated appearance, presented at our hospital with jaundice and complaining of epigastric pain. She had no history of abdominal surgery, but 6 years prior to presentation had been diagnosed with hypertension and pulmonary tuberculosis. Clinical examination found left upper quadrant pain on palpation and blood testing showed an increase in plasma bilirubin (5.09 mg/dL) in which direct bilirubin was dominant (4.71 mg/dL), aspartate aminotransferase (AST) was 75 U/L, alanine aminotransferase (ALT) was 84 U/L, cancer antigen 19‐9 (CA 19‐9) was 71.06 IU/mL, and carcinoembryonic antigen (CEA) was 1325 ng/mL.

**Figure 1:**
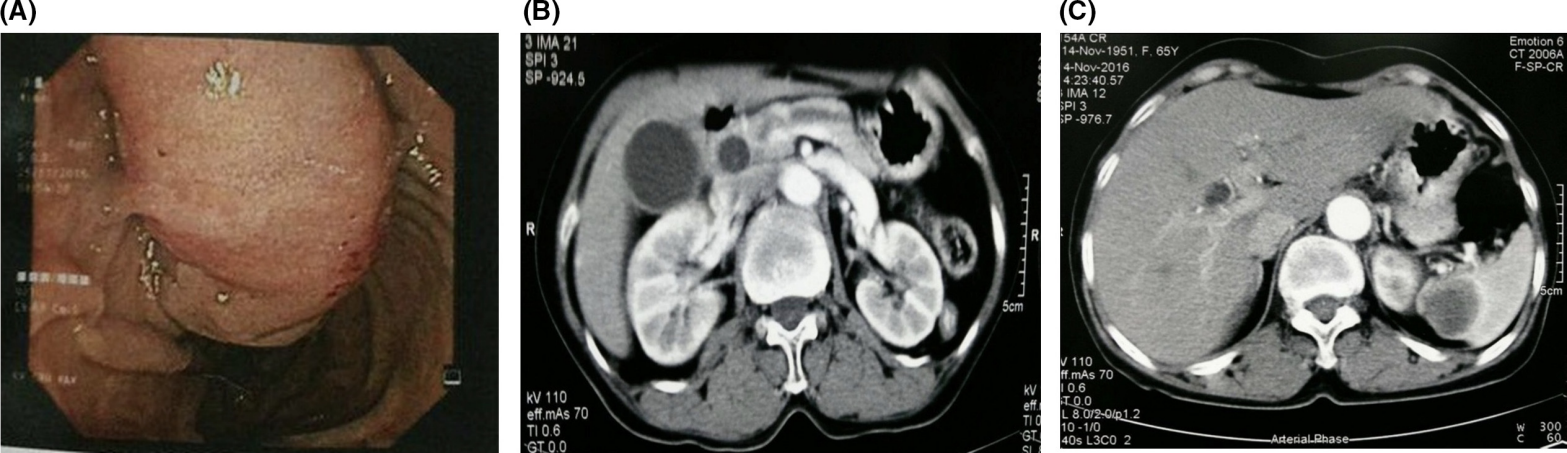
Image diagnostics before surgery. (A) The duodenoscopy revealed an ampullary tumor. (B) The abdominal computed tomography scan images showed dilation of both the internal and external hepatobiliary tract; Wirsung's diameter was 5 mm. (C) The hypodense lesion in the spleen was 3 cm.

An ampullary tumor (Fig. 1A), the dilation of common bile duct and main pancreatic duct (Fig. 1B) and a suspicious splenic mass‐related malignancy (Fig. 1C) were explored by duodenoscopy and abdominal computed tomography scan. There was no signal of invading to the superior mesenteric vein and portal vein.

Pancreatoduodenectomy and splenectomy were performed. An ampullary tumor (2 × 2 cm) and a hard splenic tumor (3 × 3 cm) were surgically resected. Again, there was no sign of any invasion to the superior mesenteric vein and portal vein or any peritoneal metastasis. The operation took 7 h with a 700 mL perioperative blood transfusion for 200 mL of blood loss. On the first day after the operation, the patient could sit up with a little wound pain, and the nasogastric tube and Foley catheter were withdrawn. On the second day, the patient was encouraged to take some food and drink because she had flatus, and the pancreatic duct drainage released approximately 100 mL of fluid.

**Figure 2:**
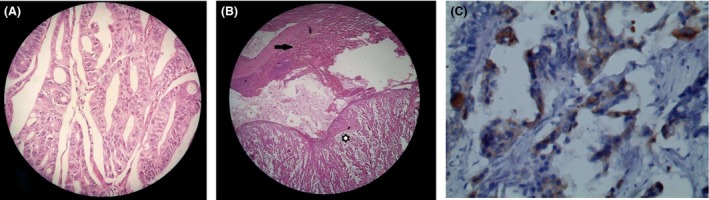
Histopathology confirmed ampullary carcinoma metastasizing to the spleen. (A) Maturation medium in histodifferentiation of ampullary carcinoma. (B) The normal splenic tissue (black arrow region) and the splenic metastasized carcinoma (asterisk region). (C) The splenic tissues with immunostaining (brown) for cytokeratin 7.

Histopathology confirmed ampullary carcinoma metastasizing to the spleen, maturation medium in histodifferentiation, invading to the duodenum and pancreas, and metastasizing to the hilus hepatis and superior mesenteric arteric lymphatic nodes, but no malignant cells were detected at border of the dissection (Fig. 2A,B). The immunohistochemical expression of cytokeratin (CK) 7 was positive in the splenic tumor while the CK 20 was negative (Fig. 2C).

The patient was discharged from the hospital 7 days after the operation without any complication and received a course of outpatient chemotherapy.

## Discussion

Clinical decision‐making is difficult in cases of this rarity where the metastatic mechanism is not fully elucidated. Hellman and colleagues 8 reported the concept of oligometastasis in 1995, namely, a medial status (≤5 metastasis) between the primary tumor and the metastases. The clinical significance of this concept is that, if the primary tumor is dissected and the metastasized tumor is also controlled by surgery or radiotherapy, it would increase the survival time and even the possibility of cure.

In our patient with a solitary splenic tumor, performing a splenectomy alongside the pancreatoduodenectomy helped to decrease the total number of cancer cells and avoid the spread of cancer cells in the blood stream or along the peritoneum. Splenectomy, indicated by the oligometastatic state of the solitary metastasis, was, therefore, a very appropriate treatment option.

## Conclusion

Here, we have described the first case to be reported of a patient with ampullary cancer with solitary metastasis of the spleen, which was successfully treated with pancreatoduodenectomy and splenectomy and was discharged 7 days after the operation with outpatient chemotherapy. Despite its rarity, ampullary carcinoma should be added to the list of possible primary tumor locations for secondary splenic metastasis. In such cases, physicians should consider splenectomy as an effective treatment option.

## Consent for Publication

Our Institute's (Department of Surgery, University of Medicine and Pharmacy, Ho Chi Minh City, Vietnam) representative was fully aware of this submission, and this scientific activity including writing manuscript was approved by the Ethic Committee of Cho Ray hospital, where the patient was operated.

## Competing Interests

None declared.

## Authorship

TMP: patient management, surgical treatment, wrote, and submitted the manuscript.
